# Cerebral Artery Alpha-1 AR Subtypes: High Altitude Long-Term Acclimatization Responses

**DOI:** 10.1371/journal.pone.0112784

**Published:** 2014-11-13

**Authors:** Ravi Goyal, Dipali Goyal, Nina Chu, Jonathan Van Wickle, Lawrence D. Longo

**Affiliations:** 1 Center for Perinatal Biology, Department of Basic Sciences, School of Medicine, Loma Linda University, Loma Linda, California, United States of America; 2 Epigenuity LLC, Loma Linda, California, United States of America; Temple University, United States of America

## Abstract

In response to hypoxia and other stress, the sympathetic (adrenergic) nervous system regulates arterial contractility and blood flow, partly through differential activities of the alpha1 (α_1_) - adrenergic receptor (AR) subtypes (α1A-, α1B-, and α1D-AR). Thus, we tested the hypothesis that with acclimatization to long-term hypoxia (LTH), contractility of middle cerebral arteries (MCA) is regulated by changes in expression and activation of the specific α_1_-AR subtypes. We conducted experiments in MCA from adult normoxic sheep maintained near sea level (300 m) and those exposed to LTH (110 days at 3801 m). Following acclimatization to LTH, ovine MCA showed a 20% reduction (n = 5; P<0.05) in the maximum tension achieved by 10^−5^ M phenylephrine (PHE). LTH-acclimatized cerebral arteries also demonstrated a statistically significant (P<0.05) inhibition of PHE-induced contractility in the presence of specific α1-AR subtype antagonists. Importantly, compared to normoxic vessels, there was significantly greater (P<0.05) α1B-AR subtype mRNA and protein levels in LTH acclimatized MCA. Also, our results demonstrate that extracellular regulated kinase 1 and 2 (ERK1/2)-mediated negative feedback regulation of PHE-induced contractility is modulated by α1B-AR subtype. Overall, in ovine MCA, LTH produces profound effects on α1-AR subtype expression and function.

## Introduction

Acute hypoxia leads to a significant increase in cerebral blood flow [1]. However, with successful acclimatization to hypoxia, the cerebral blood flow returns to the values similar to individuals at sea-level [2], [3]. Associated changes with the normal acclimatization response include: hypercapnia, polycythemia, high hemoglobin concentration, and angiogenesis. These changes are crucial to maintain normal blood flow normal with adequate tissue oxygenation [4]. Dysregulation of the normal acclimatization responses can lead to acute or chronic mountain sickness, high altitude cerebral edema, chronic migraine headache, and other high altitude-associated disorders [5]–[8]. To study the cellular/sub-cellular mechanisms responsible for successful acclimatization, we exposed adult sheep to LTH (∼110 days) at an altitude of 3801 m. In previous studies, at this altitude, we have reported that the arterial PO_2_ fell by 40% and cardiac output decreased 14% [9], [10]. Of note, in the LTH animal the distribution of the reduced cardiac output was altered so that blood flow to the brain was maintained at near normal levels [2], [3].

Thus, to maintain cerebral blood flow despite a steady-state decrease in cardiac output, the basal cerebrovascular resistance probably decreases in response to acclimatization to LTH. One possible contribution to a decrease in cerebrovascular resistance could be a shift in the structure and/or composition of the cerebral arteries favoring larger diameters and reduced hydraulic resistance. Inconsistent with this possibility, LTH had no significant effects on average artery wall thicknesses or water content [11].

Another mechanism that could potentially contribute to the LTH associated decreased cerebrovascular resistance is that of decreased vascular tone. Responses to exogenous nitric oxide (vasodilator) released from s-nitroso-N-acetylpenicillamine, however, does not differ significantly in normoxic and LTH sheep MCA [3]. Similarly, LTH had no significant effect on vasodilator responses to the calcium ionophore A23187 or shear-stress-induced nitric oxide release in fetal MCA [3]. Thus, based on our previous studies, clearly the reduced cerebrovascular resistance characteristic of LTH acclimatization must involve other mechanisms.

Another important influence on cerebrovascular resistance under stress is the release of vasoactive neurohormones from perivascular nerves. The largest neural component of the cerebral vasculature is adrenergic in nature [12]–[15], and this serves an important role in regulating cerebral arterial contractility and blood flow [13], [16]. Importantly, the role of adrenergic regulation increases substantially during stress, and plays an important role in maintaining cerebral blood flow [17]. Also, acclimatized sheep have significantly higher basal norepinephrine and epinephrine levels compared to sea-level controls [11]. Paradoxically, despite these increased catecholamine levels, we observed ∼20% reduction in contractile responses to nor-epinephrine in LTH acclimatized sheep cerebral arteries [11]. To explore the mechanisms of these findings, we examined α1-AR densities on the sea-level normoxic control and LTH acclimatized arteries. With LTH acclimatization, we observed a 66% and 61% reduction in α1-AR density in sheep common carotid and MCA, respectively [18].

Radio-ligand binding and molecular cloning in several species have demonstrated that the α1-AR family has three structurally distinct subtypes (α1A-, α1B-, α1D-), which are widely expressed in tissues including cerebral arteries, and have differing amino acid sequences and pharmacological properties [19]. Several reviews have considered these in detail [20]–[22]. Although the three α_1_-adrenoceptor subtypes have been reported in various cell types, little is known about their expression, physiological functions, or downstream pathways in MCA. We have shown that in adult ovine cerebral arteries all three α1-AR subtypes are present [12]. Unknown, however, is the extent to which these subtypes are modulated as a consequence of LTH. Thus, we tested the hypothesis that LTH acclimatization of cerebral arterial contractility is regulated by changes in expression and/or activation of α_1_-AR subtypes (α_1A_-, α_1B_-, and α_1D_-AR). The present study provides a deeper understanding of cerebral arterial contractility, and provides insights into the LTH regulation of α1-AR expression.

## Methods

### Experimental animals and tissues

All experimental procedures were performed within the regulations of the Animal Welfare Act, the National Institutes of Health Guide for the Care and Use of Laboratory Animals, and were approved by the Animal Care and Use Committee of Loma Linda University. Sheep were obtained from Nebeker Ranch (Lancaster, CA), as previously described [12], [23]. For these studies, we used MCA from ∼2 years old ewes that either had been maintained near sea level (300 m) or those acclimatized to high altitude (3,801 m, 12,470 ft; Barcroft Laboratory, White Mountain Research Station, Bishop, CA) for ∼110 days immediately before the studies.

For every experiment, 4 to 5 sheep were used from each experimental group. A total of 30 normoxic controls and 30 LTH sheep were used for completion of the present study. Following LTH exposure, animals maintained at high altitude were transported to the Center for Perinatal Biology, Loma Linda University. Upon their arrival at the Center, we placed a tracheal catheter in the sheep through which N_2_ flowed at a rate adjusted to maintain its PaO_2_ at ∼60 Torr, similar to the value at high altitude [24]. All studies were conducted within 48 hours of placing the tracheal catheter. At the time of study, ewes were euthanized with an overdose of the proprietary solution, Euthasol (pentobarbital sodium 100 mg/Kg and phenytoin sodium 10 mg/Kg; Virbac, Ft. Worth, Tx). The brains were removed, following which we obtained the MCA for further analysis. We performed studies in isolated vessels cleaned of adipose and connective tissue. To avoid the complications of endothelial-mediated effects, we carefully removed the endothelium by inserting a small wire three times, as described previously [12], [23]–[26]. Removal of endothelium was examined functionally by stimulating the arterial segments with 122 mM KCl and at the plateau of the response, 100 µM acetylcholine was applied [24], [27]. Failure of acetylcholine to relax the arterial segment was taken as a confirmation of the removal of endothelium. On application of acetylcholine, if the arterial segment relaxed, it was discarded from the study. We used the vessels immediately for the experiments. Table 1 presents the specific pharmacological agents used in the present study.

**Table 1:** Pharmacological Agents Used in the Present Study.

Full Name	Abbreviation	Conc.Molar	Target	Company	Cat #
PHE HCl	PHE	10^−5^	Α1-ARantagonist	Sigma	P-6126
2-([2,6-Dimethoxyphenoxyethyl]a-minomethyl)-1,4-benzodioxane hydrochloride	WB	10^−7^	α1A-ARantagonist	Sigma	B-018
chlor-ethyl-clonidine	CEC	10^−5^	α1B-ARantagonist	Sigma	B-003
8-(2-[4-(2-Methoxyphenyl)-1-piperazinyl]ethyl)-8-azaspiro(4.5)decane-7,9-dione	BMY	10^−7^	α1D-ARantagonist	Sigma	B-134
	PD98059	2×10^−5^	ERK inhibitor	Sigma	P-215
Y-27632 dihydrochloride	Y27632	10^−5^	Rho-Kinaseinhibitor	Tocris	1254
2-[1-(3-(Amidinothio)propyl)-1H-indol-3-yl]-3-(1-methylindol-3-yl)maleimide methanesulfonate	Ro31-8220	10^−5^	Pan-PKCinhibitor	Enzo LifeSciences	EL-283
2,2',3,3',4,4'-Hexahydroxy-1,1' -biphenyl-6,6'-dimethanol-dimethyl ether	HBDDE	5×10^−5^	PKCα+PKCγinhibitor	Enzo LifeSciences	EL-273
4,5-bis[(4-Fluorophenyl)amino]phthalimide	DAPH-7	4×10^−6^	PKCβ inhibitor	Enzo LifeSciences	EL-387
N-Myristoyl-Glu-Ala-Val-Ser-Leu-Lys-Pro-Thr	MyristoylatedPKC eV-2	10^−5^	PKCε inhibitor	Biomol LifeSciences	P-223

### Measurement of middle cerebral artery contractility

We have described this technique in several reports [12], [23], [24], [28]–[30]. MCA were dissected free from parenchyma and cut into 5 mm long rings in ice-cold modified Krebs-Henseleit (K–H) solution at 4–5°C containing in mM: 120 NaCl; 4.8 KCl; 1.2 K_2_HPO_4_; 25 NaHCO_3_; 1.2 MgCl_2_; 2.5 CaCl_2_; 10 glucose. The arterial rings were suspended in organ baths (Radnoti Glass Instruments, Inc. Monrovia, CA) that contained 10 ml of modified K-H buffer maintained at 37°C and aerated with 95% O_2_ and 5% CO_2_ (pH = 7.4). Isometric force was recorded using low compliance force transducers (Radnoti Glass Instruments, Inc) with analogue to digital data collection systems and software (Powerlab 16/30/Chart 5.5 AD Instruments, Colorado Springs, CO). At the beginning of each experiment, vessels were equilibrated without tension for one hour. In view of our previous findings, following stabilization the optimum resting tension was 0.7 gram (g), as at this tension the contractility response to 122 mM KCl was maximal. The contractile response to 122 mM KCl was used to normalize for the variation in smooth muscle mass in each arterial ring [12], [23]. For all vessels, we evaluated the contractile response for tension by measuring the maximum peak height, and expressing it in both absolute terms and as percentage K_max_ (a measure of “efficacy”), and calculated pD_2_ (the negative logarithm of the EC_50_, or half-maximal concentration for PHE (a selective α_1_-AR agonist), and an index of tissue “sensitivity” or “potency”).

### The Role of α_1_-AR subtype blockers

Three pharmacologically distinct α_1_-AR subtypes can be distinguished with pharmacological agents [12], [19]. The α_1A_-AR is selectively inhibited by WB [2-([2,6-Dimethoxyphenoxyethyl]a-minomethyl)-1,4-benzodioxane hydrochloride] [31], the α_1B_-AR subtype is selectively inhibited by CEC (chlor-ethyl-clonidine) [32], and BMY [8-(2-[4-(2-Methoxyphenyl)-1-piperazinyl]ethyl)-8-azaspiro(4.5)decane-7,9-dione] selectively inhibits the α_1D_-AR subtype [33]. Each of these antagonists is well characterized with known inhibitory concentration to reduce 50% of PHE response (IC_50_) for vascular adrenergic receptors. First, using a given concentration of PHE (10^−9^ to 10^−2^ M) PHE, we performed a dose-response curve. Then following washout (∼5 min) and 40 min re-equilibration, we repeated the PHE dose-response in the presence of the α_1_-AR subtype blockers (concentrations are given in Table-1). To examine the role of α1-adrenergic receptor subtypes, the antagonists were administered 20 min before the application of PHE. For each agent, we determined the IC_50_ and dissociation constant K_B_ (Table 2)_._

**Table 2:** Inhibitory concentration for 50% reduction in PHE contractile responses (IC_50_) and Dissociation constants (-K_B_) values of α_1_-AR subtypes inhibitors for phenylephrine-induced contractility in ovine middle cerebral arteries.

Receptor-Drug	K_B_ (M)	IC_50_ - Values
α1A – WB (10^−7^ M)	1.1×10^−8^	6.9±0.1
α1B – CEC (10^−5^ M)	5.3×10^−10^	7.3±0.1
α1D – BMY (10^−7^ M)	6.7×10^−9^	6.5±0.1

### Immunoblot of α_1_-AR subtypes

As noted, ovine MCA were cleaned of adventitia and the endothelium was denuded in a phosphate-free balanced salt solution (BSS) of the following composition (mM): 126 NaCl; 5 KCl; 10 HEPES; 1 MgCl_2_; 2 CaCl_2_; 10 glucose; pH 7.4 (adjusted with NaOH). The arteries were homogenized with a tissue grinder in ice-cold cell lysis buffer (Cell Signaling Technology, Danvers, MA), as described previously [12]. Protein concentrations were measured using a protein assay kit (Bio-Rad Laboratories, Hercules, CA) and bovine serum albumin (BSA) was used as a reference protein standard. Mini Trans-Blot Electrophoretic Transfer Cell system (Bio-Rad Laboratories) was used to transfer proteins from the gel to a nitrocellulose membrane at 100 V for 3 h. We then performed an overnight incubation of subtype specific primary antibodies (1∶500 dilution) for α1A-, α1B-, and α1D-AR (Santa Cruz Biotechnology, Santa Cruz, CA). We used α-actin as an internal control for equal protein loading, as well as the blocking peptide for each subtype specific antibody as a negative control (Santa Cruz Biotechnology). The membrane was then incubated in chemiluminescence luminol reagent (Pierce, Rockford, IL) for 1 min, and the protein band was detected using Alpha Innotech Chemiluminescent imaging system (San Leandro, CA).

### Real Time PCR Analysis of mRNA Expression

To examine the extent to which LTH induced changes in α1-AR subtype mRNA expression, we examined the expression levels of the three subtypes by real-time PCR. We designed primers with the use of Primer 3 web-based software (http://frodo.wi.mit.edu/primer3/). These primers (Table 3) were synthesized by Integrated DNA technologies (Coralville, CA). We isolated and quantified RNA by Allprep DNA/RNA Mini Kit, according to the manufacturer’s instructions (Qiagen Inc, Valencia, CA Cat # 80204). To check for RNA quality and quantity, isolated mRNA was analyzed using a NanoDrop 1000 Spectrophotometer (Thermofisher Scientific, Waltham, MA) at 260/280 wavelength UV rays, and a 260/280 ratio of 1.8 to 2 was accepted for quantification with real-time PCR. RNA integrity was analyzed on 1% agarose gel (Figure S2). Samples with clear 28 s and 18 s bands were included in further analysis. The mRNA was treated with genomic DNA wipeout buffer, and then reverse transcribed using Quantitect cDNA synthesis kit (Qiagen, Valencia, CA). Relative expression was normalized to 18S RNA, and fold-changes were calculated using the ΔΔCt method with normalization of individual PCR efficiencies [34]. GAPDH and Beta Actin also were examined as controls (Figure S1A & S1B). Samples (n = 4 from each group) were analyzed on the Roche LightCycler 1.5 (Roche, Indianapolis, IN).

**Table 3:** Primers used in the present study.

Gene Name	Accession No.	Forward Primer	Reverse Primer	Amplicon Length (Base Pairs)
ADRA1A	XM_004004166.1	TGCACTCGGTCACACACTAC	TGACTTGTCGGTCTTGAGGC	492
ADRA1B	XM_004009542.1	GAAGAAGACCACGGGGGAAG	AGAAGGGCAGAACGGTGAAG	411
ADRA1D	XM_004014355.1	CAACGTGCTGGTCATCCTCT	ATAGCCTGCCTCCTCTGTGA	424
18S	AY753190.1	GCTCGCTCCTCTCCTACTTG	GATCGGCCCGAGGTTATCTA	190
GAPDH	NM_001190390.1	GAGCGAGATCCTGCCAACAT	GAAGTCGCAGGAGACAACCT	624
ACTB	NM_001009784.1	ATGCTTCTAGGCGGACTGTT	CGAAGACAGGAGCAGTGGAG	248

### Statistical analysis

All values were calculated as means ± standard error of mean (SEM). In all cases, *n* values refer to the number of animals for a particular study in each experimental group. For testing differences between two groups, we used a simple unpaired Student’s t-test. Where applicable, we used analysis of variance with repeated measures (Prism, GraphPad Software, La Jolla, CA). A *P*-value of <0.05 was considered significant.

## Results

### Changes in PHE response with LTH acclimatization

**Figure 1:**
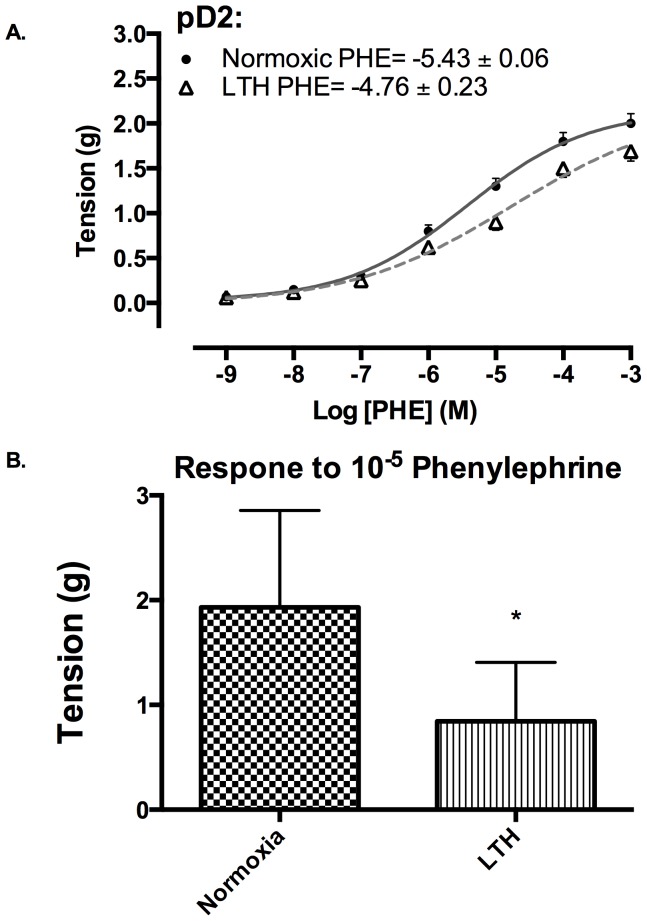
Phenylephrine (PHE) responses in normoxic and LTH ovine MCA. (*A*) Dose-response curves in response to PHE under normoxic (•, solid line) and LTH (Δ, dashed line) conditions. (*B*) Tension (g) at EC_50_ in normoxic and LTH sheep MCA. n = 5 sheep in each group. Values are means ± standard error of means. *Denotes P = <0.05.

With LTH acclimatization, we observed a right-shift of the PHE dose-response curve (Figure 1A). The EC_50_ for PHE was ∼10^−5^ M in both oxygenation groups. Also, the maximum tension achieved in MCA by 10^−5^ M PHE was reduced significantly in the LTH exposed arteries as compared to the normoxic control (Figure 1B).

### Role of α_1A_-AR subtypes in cerebrovascular contraction

**Figure 2:**
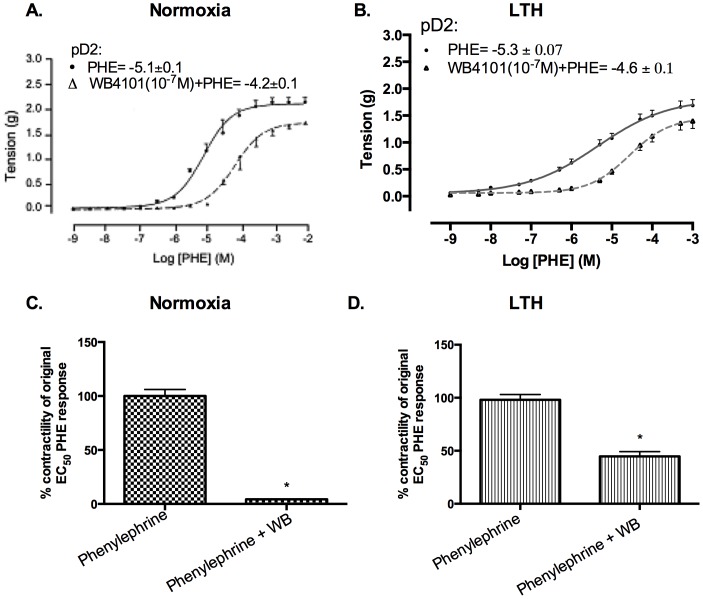
PHE responses in presence and absence of α1A-AR subtype antagonist (10^−7^ M WB) in normoxic and LTH ovine MCA. (A) Dose-response curves in normoxic MCA. Vascular tensions (g) for MCA in response to PHE alone (•, solid line), and in the presence of 10^−7^ M WB (Δ, dashed line).(B) Dose-response curves in LTH MCA. (C) Tension (g) at EC50 in normoxic sheep MCA. (D) Tension (g) at EC50 in LTH sheep MCA. n = 5 sheep in each group. Values are means ± standard error of means. *Denotes P = <0.05.

In both normoxic and LTH ovine MCA (5 animals in each group), the α1A-AR subtype antagonist (WB) reduced the PHE-induced contractility (Figure 2A & B). In normoxic animals, in the presence of α1A-AR antagonist (WB4101, 10^−7^ M) the receptor sensitivity for PHE was reduced ∼20% (pD2 increased from –5.1±0.1 to –4.2±0.1). In LTH MCA, however, the α1A-AR antagonist reduced the receptor sensitivity for PHE only ∼15% (pD2 increased from –5.3±0.1 to –4.5±0.06). Importantly, in normoxic cerebral arteries 10^−7^ M WB reduced 10^−5^ M (EC_50_) PHE-induced contractile response ∼90% (Figure 2C). While, in LTH acclimatized arteries, the 10^−7^ M of WB only reduced the 10^−5^ M PHE-induced contractile responses ∼50% (Figure 2D). Thus, it appears that the role of α1A-AR subtype is reduced in PHE-induced contractility.

### α_1A_-AR expression

**Figure 3:**
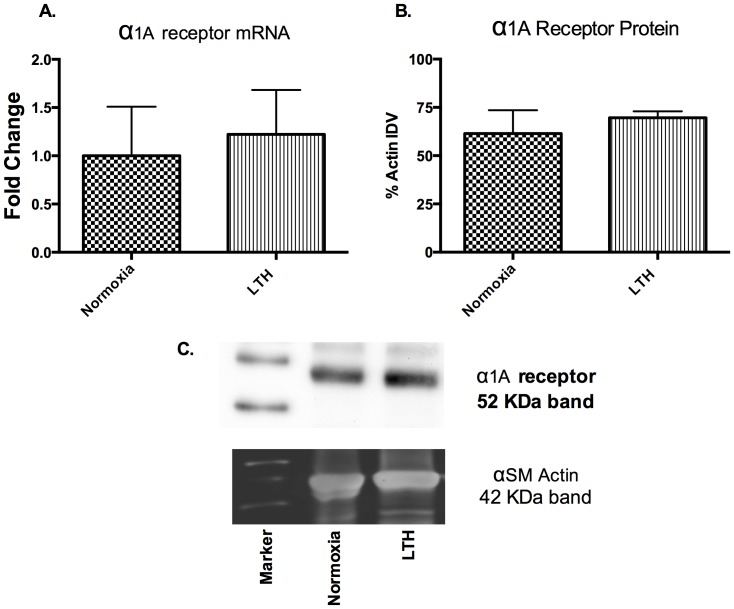
α1A AR expression. (A) Relative mRNA levels in normoxic and LTH cerebral arteries by real-time PCR. (B) Relative α1-AR antigen levels detected by western immunoblot analysis. IDV - Integrated Density Value. n = 5 sheep in each group. Values are means ± standard error of means. *Denotes P = <0.05. Fold change was relative to 18 s Ribosomal RNA.

The reduced role of α1-AR subtype in PHE-induced contractility may be a consequence of reduced expression. Therefore, we examined mRNA and protein levels of the α1A-AR subtype. Neither mRNA and nor protein levels of α1A-AR subtype expression were changed with LTH (Figure 3A, B, & C).

### Role of α_1B_-AR subtypes in cerebrovascular contraction

**Figure 4:**
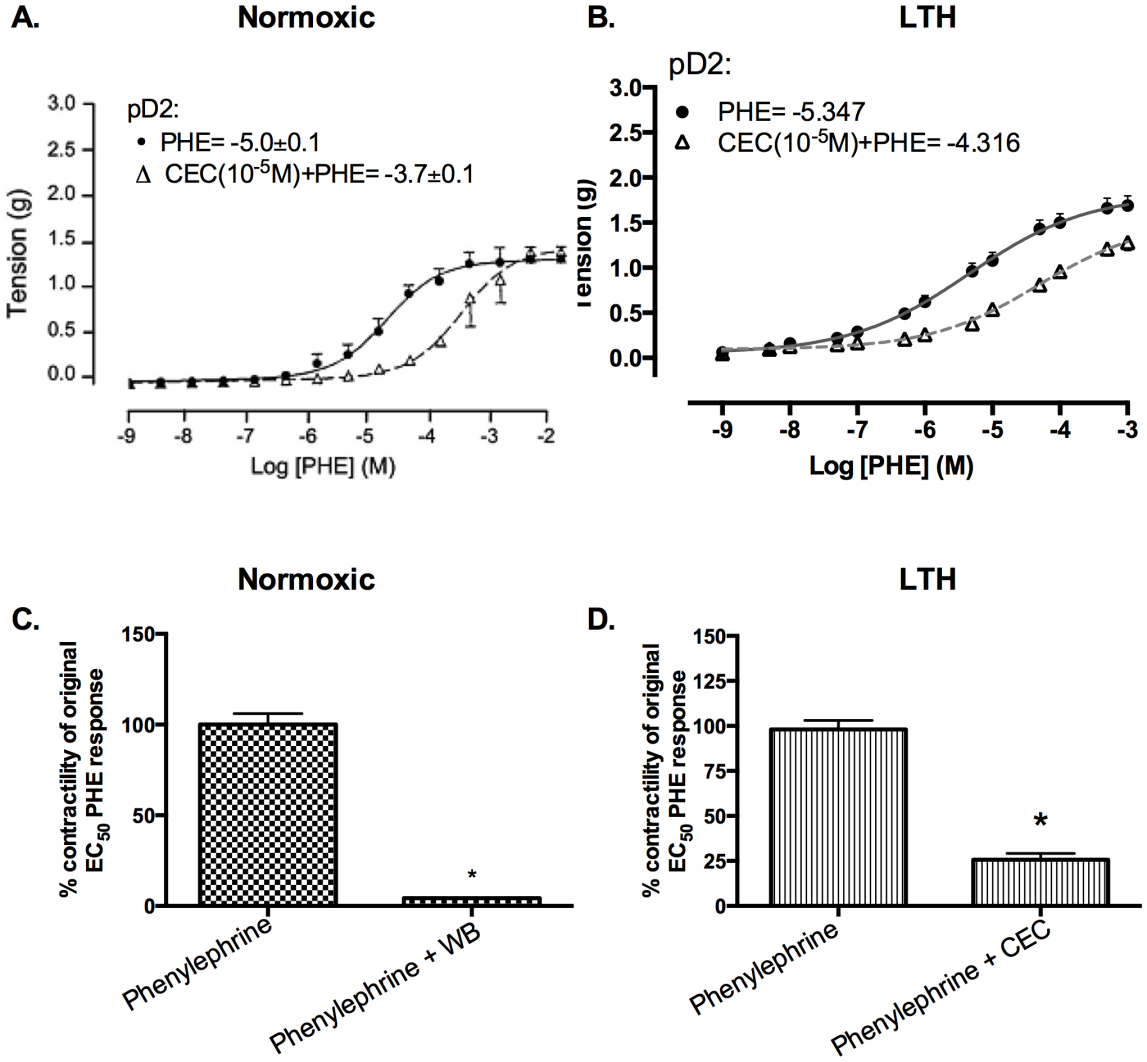
PHE responses in presence and absence of α1B-AR subtype antagonist (10^−5^ M CEC) in normoxic and LTH ovine MCA. (A) Dose-response curves in normoxic MCA. Vascular tensions (g) for MCA in response to PHE alone (•, solid line), and in the presence of 10^−5^ M CEC (Δ, dashed line).(B) Dose-response curves in LTH MCA. (C) Tension (g) at EC50 in normoxic sheep MCA. (D) Tension (g) at EC50 in LTH sheep MCA. n = 5 sheep in each group. Values are means ± standard error of means. *Denotes P = <0.05.

As seen in Figure 4A & B, selective α1B-AR antagonist (10^−5^ M CEC) reduced the receptor PHE sensitivity ∼26% (pD2 increased from –5.0±0.1 to –3.7±0.1) and 19% (pD2 increased from –5.3±0.1 to –4.3±0.1) in normoxic and LTH MCA, respectively. Additionally, 10^−5^ M of CEC, reduced the 10^−5^ M PHE induced contractility ∼85% and ∼74% in normoxic and LTH MCA, respectively (Figure 4C & 4D). Thus, LTH resulted in a significant reduction in the role of the α1B-AR subtype in PHE-induced contractility.

### α_1B_-AR expression

**Figure 5:**
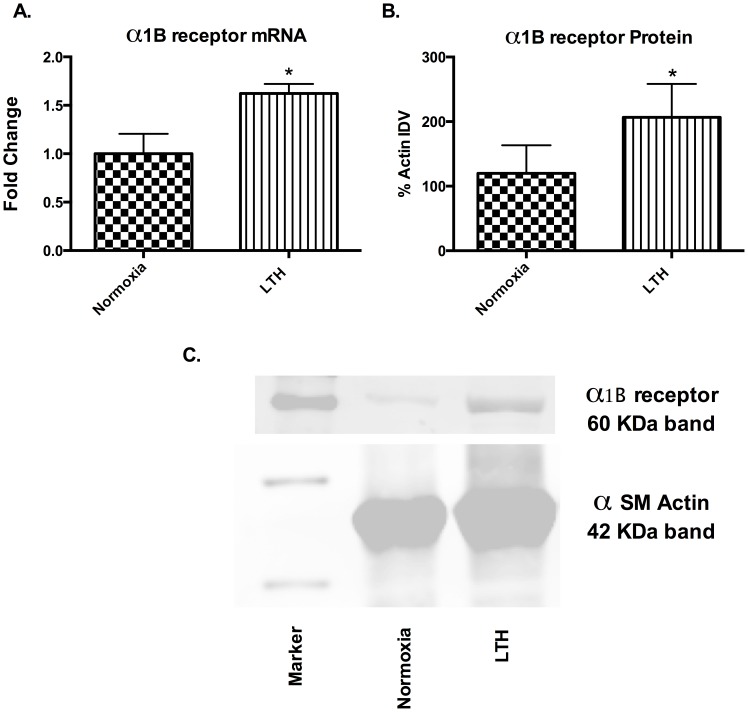
α1B AR expression. (A) Relative mRNA levels in normoxic and LTH cerebral arteries by real-time PCR. (B) Relative α1-AR antigen levels detected by western immunoblot analysis. IDV - Integrated Density Value. n = 5 sheep in each group. Values are means ± standard error of means. *Denotes P = <0.05. Fold change was relative to 18 s Ribosomal RNA.

As noted above, the reduced role of the α1B-AR subtype in PHE-induced contractility may be a consequence of reduced expression. Thus, we examined the α1B-AR mRNA and protein levels. Surprisingly, both mRNA and protein levels of α1B-AR subtype increased significantly with LTH acclimatization (Figure 5A, B, & C).

### Role of α_1D_-AR subtypes in cerebrovascular contraction

**Figure 6:**
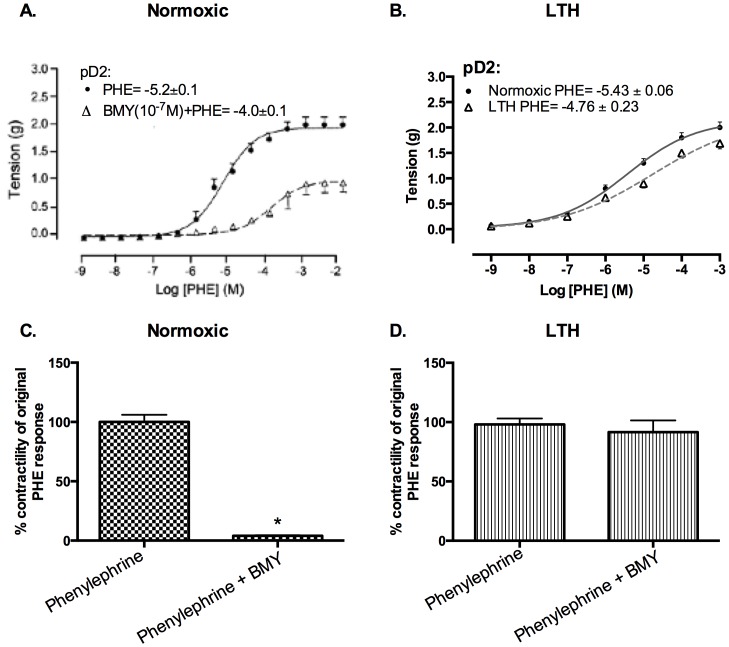
PHE responses in presence and absence of α1D-AR subtype antagonist (10^−7^ M BMY) in normoxic and LTH ovine MCA. (A) Dose-response curves in normoxic MCA. Vascular tensions (g) for MCA in response to PHE alone (•, solid line), and in the presence of 10^−7^ M BMY (Δ, dashed line).(B) Dose-response curves in LTH MCA. ((C) Tension (g) at EC50 in normoxic sheep MCA. (D) Tension (g) at EC50 in LTH sheep MCA. n = 5 sheep in each group. Values are means ± standard error of means. *Denotes P = <0.05.

Next, we examined the role of α1D-AR in PHE-induced contractile responses. As demonstrated in Figure 6A, in normoxic MCA, the α1D-AR antagonist (BMY) produced a significant ∼95% reduction in 10^−5^ PHE-induced contractile response. In contrast, in the MCA from LTH acclimatized animals, α1D-AR antagonist reduced the 10^−5^ M PHE-induced contractile response only ∼9% (Figure 6B). Importantly, the changes in PHE receptor sensitivity in the presence of α1D-AR antagonist were ∼24% (pD2 increased from –5.2±0.1 to –4±0.1) and ∼–3% (pD2 increased from –5.3±0.1 to –5.5±0.05) in normoxic and LTH MCA, respectively, and as with α1A-AR subtype contractility was reduced to a much lesser extent in LTH, compared to normoxic vessels (Figure 6C & 6D).

### α_1D_-AR expression

**Figure 7:**
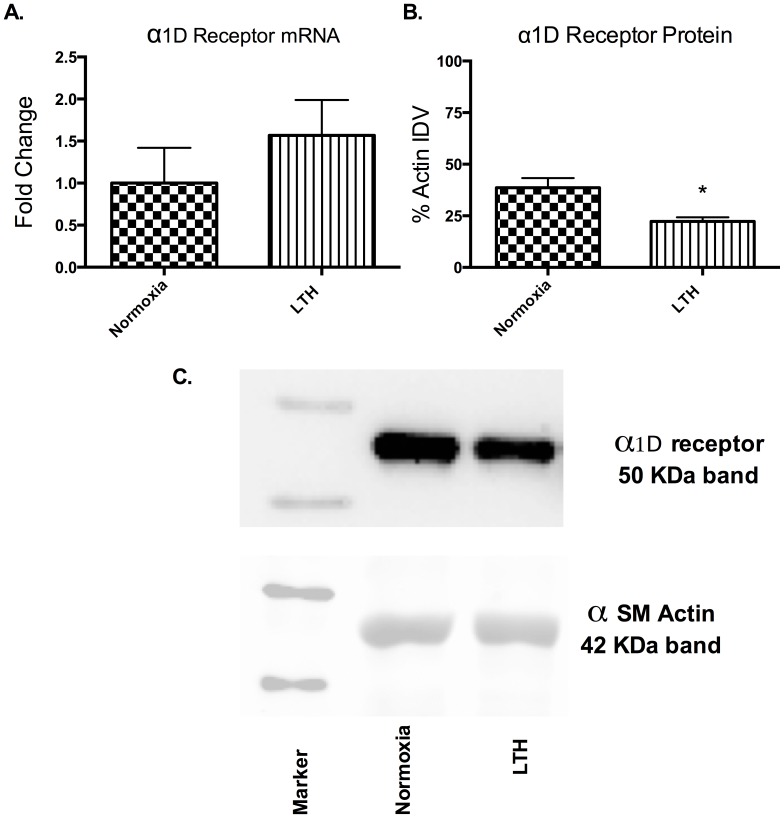
α1D AR expression. (A) Relative mRNA levels in normoxic and LTH cerebral arteries by real-time PCR. (B) Relative α1-AR antigen levels detected by western immunoblot analysis. IDV - Integrated Density Value. n = 5 sheep in each group. Values are means ± standard error of means. *Denotes P = <0.05. Fold change was relative to 18 s Ribosomal RNA.

Similar to the reduced role of α1D-AR subtype in contractile responses, while we observed no change in α1D-AR mRNA levels (Figure 7A) there was a ∼30% decrease in its protein levels (Figure 7B & C).

LTH-induced changes in contractile responses did not co-relate with the changes in receptor expression. Thus, LTH may be regulating the α1-AR mediated contractile responses by downstream signal transduction pathways. A major mechanism through which α1-AR regulates contractile responses is through protein kinase C (PKC).

### LTH and role of PKC in PHE-mediated contractility

**Figure 8:**
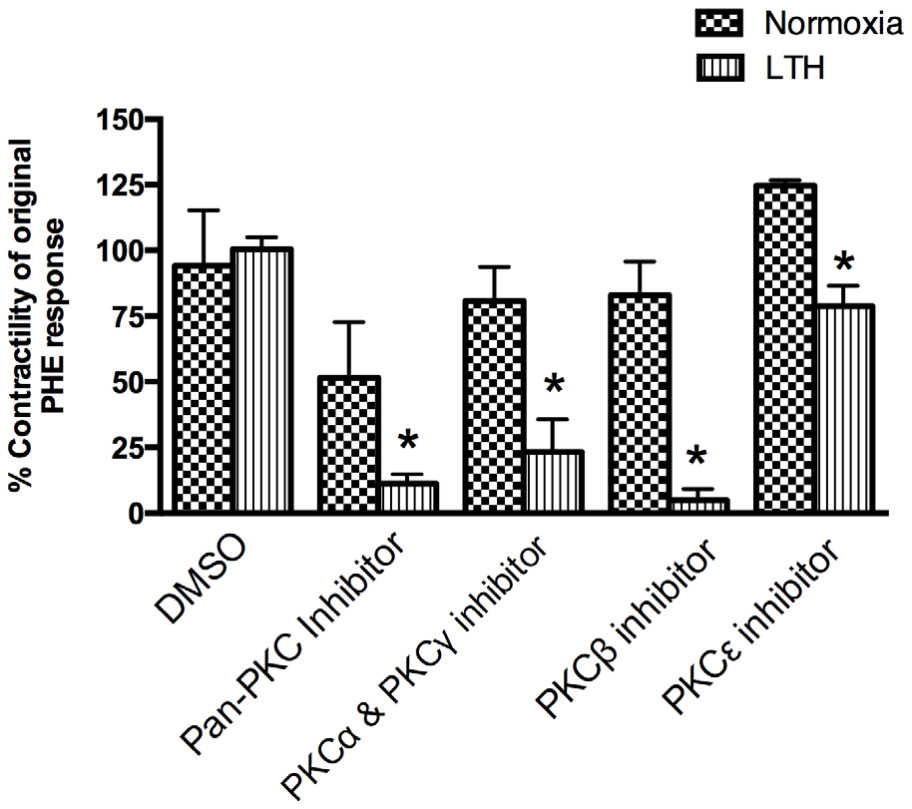
PHE responses in the presence of PKC inhibitors. DMSO - Dimethyl sulfoxide was used as vehicle for PKC inhibitors. Pan-PKC inhibitors –10^−5^ M Ro31-8220; PKCα and γ inhibitor –5×10^−5^ M HBDDE, PKCβ inhibitor –4×10^−6^ M DAPH7, and PKC epsilon –10^−5^ Myristoylated PKC eV1-2 inhibitor. n = 5 sheep in each group. Values are means ± standard error of means. *Denotes P = <0.05.

Figure 8 demonstrates the relative role of PKC and several of its isoforms in the two experimental groups. As evident, with LTH exposure, MCA demonstrated a significant increase in PKC antagonist-mediated inhibition of PHE induced contractility. The major PKC isoforms appear to be PKC α, β and γ.

### LTH and role of Extracellular Regulated Kinases 1 and 2 (ERK1/2) in PHE-mediated contractility

**Figure 9:**
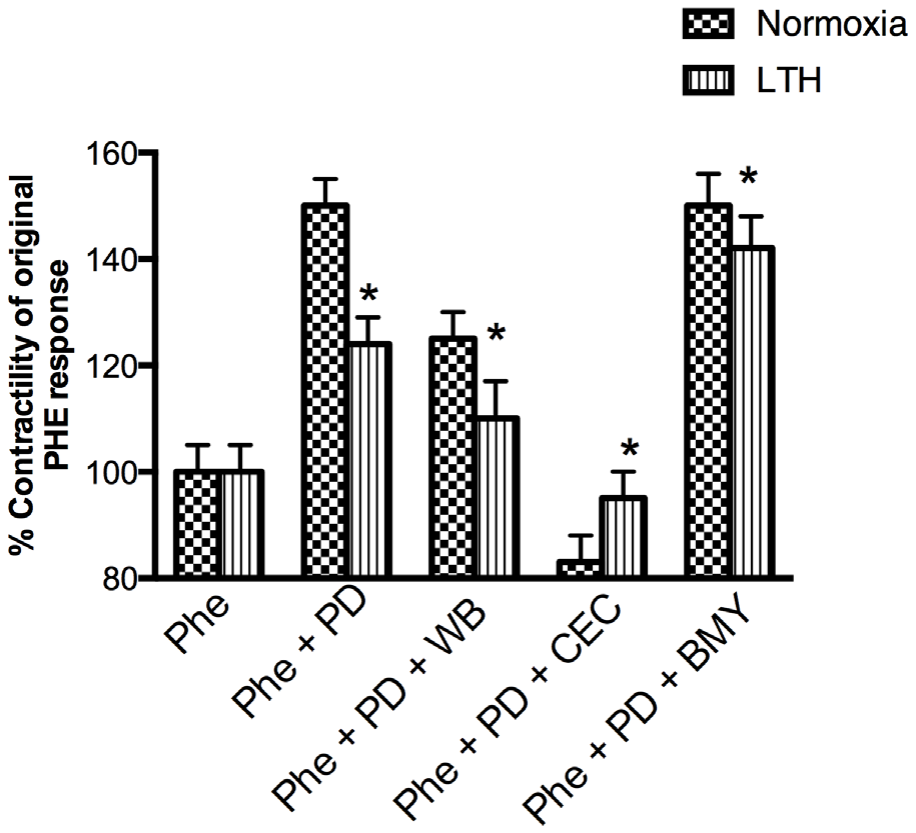
PHE responses in the presence of ERK inhibitors in normoxic (A) and LTH (B) ovine MCA. Specific α1-AR antagonists and ERK inhibitor was added 20 minutes before adding PHE. PHE –10^−5^ PHE; ERK inhibitor - PD98059 –2×10^−5^ PD; WB - α1-AR subtype antagonist –10^−7^ M WB-4101; CEC - α1B-AR subtype antagonist –10^−5^ M chlor-ethyl-clonidine; BMY - α1D-AR subtype antagonist –10^−7^ M BMY-7378. n = 5 sheep in each group. Values are means ± standard error of means. *Denotes P = <0.05.

Previously, we have demonstrated that ERK1/2 act as negative regulators of PKC-induced contractile responses [23]. Therefore, we examined the role of ERK1/2 in PHE-induced MCA contractility under conditions of LTH. In response to the ERK1/2 antagonist (2×10^−4^ PD98059), we observed a significant increase in PHE-induced contractility in both normoxic and LTH arteries (Figure 9). However, ERK1/2 inhibition produced significantly less increase in the PHE-induced contractile responses in MCA from LTH acclimatized animals. To elucidate further the specific α1-AR subtypes mediating ERK activation, we examined the PHE-induced contractile responses in the presence of ERK inhibitor (2×10^−5^ PD98059) in presence of specific α1-AR antagonists. As seen in Figure 9, inhibition of α1B-AR subtype abolishes the ERK-mediated negative regulation of PHE contractility in the normoxic vessel, while decreasing it significantly in LTH. Of importance, inhibition of α1A- and α1D-AR receptors failed to abolish the ERK-mediated negative regulation of PHE-induced MCA contractility.

### LTH and role of Rho kinase in PHE-mediated contractility

**Figure 10:**
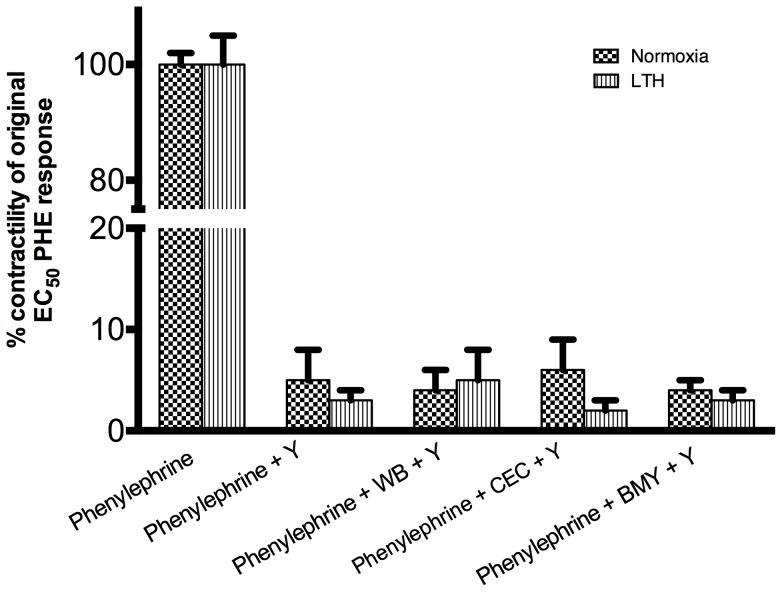
PHE responses in the presence of rho kinase inhibitor (10^−5^ M Y-27632) and specific α1-AR subtypes antagonists. n = 5 sheep in each group. Values are means ± standard error of means. *Denotes P = <0.05.

As seen in Figure 10, upon application of a Rho kinase inhibitor (10^−5^ Y-27632), we observed essentially a complete inhibition of PHE-induced contractility in both normoxic and LTH acclimatized MCA. No significant differences were observed in the presence of the several α1-AR subtype inhibitors.

## Discussion

The present report is an extension of our previous studies on the mechanisms by which the cerebral vasculature acclimatizes to LTH. Previously, we have demonstrated that in response to such acclimatization, cerebral blood flow is maintained to levels similar to those in a normoxic animal [2], [3]. Importantly, an inability to maintain normal cerebral blood flow may lead to life threatening disorders such as high-altitude associated cerebral edema and other cerebral manifestations of acute/chronic mountain sickness. Also, in the LTH acclimatized sheep, we have observed a significant increase in norepinephrine (NE) levels and a ∼20% reduction in NE-induced MCA contractile responses compared to normoxic controls [11]. The catecholamine NE can act on α1, α2, and/or β1 receptors; however, we have shown that MCA contract in response to NE via stimulation of α1-AR [12], [30]. Also, the α2-AR are chiefly pre-junctional in MCA and do not appear to regulate NE-mediated contractile responses [35].

In the present study, by use of α1-AR specific agonist (PHE) and subtype antagonists, we examined the role of LTH in mediating the regulation of specific α1-AR subtypes. In LTH acclimatized cerebral arteries, PHE-induced contractile responses were reduced to a similar extent (∼20%), as compared to norepinephrine [11]. Nonetheless, based on our previous [18] and the present study, the sensitivity of α1-AR is reduced with LTH acclimatization (Figure 1). Importantly, in the present study, we observed a significantly lower role of α1A-AR subtype in PHE-induced contractile responses, as compared to their normoxic counterpart (Figure 2). We observed no reduction in α1a-AR mRNA or protein expression (Figure 3). Therefore, it appears that the reduction in the role of α1-AR in PHE-induced contractile responses may be downstream at the second messenger level or may be negated by some counter-regulatory mechanisms such as nitric oxide pathway. Another explanation for this may be that receptors are being internalized. In a similar manner, LTH acclimatization reduced the role of α1B-AR in PHE-induced MCA contractile responses (Figure 4), despite a significant increase in both the α1B-AR mRNA and protein levels (Figure 5). A clear rationale for the inverse relationships observed is not known. In addition, we observed a significantly reduced role of α1D-AR in PHE-induced contractile responses in LTH acclimatized arteries. This was associated with a significant reduction in α1D-AR protein expression, despite no significant change in mRNA levels. This suggests a post-transcriptional regulation of α1D-AR subtype protein expression. Apparently changes in the expression of α1-AR as well as the PHE-induced contractile responses due to LTH acclimatization suggest changes in the downstream signaling pathways. As is well known, the three α_1_-AR subtypes, members of G_q_/_11_ protein coupled receptors, activate PLC-β to hydrolyze phosphatidylinositol 4,5,bisphosphate to form inositol 1,4,5, tri-phosphate [Ins(1,4,5)P_3_] and di-acyl glyerol (DAG). Ins(1,4,5)P_3_ acts on its receptors in the sarcoplasmic reticulum to trigger calcium release and other calcium dependent contractility pathways. DAG with and without calcium activates the several PKC isoforms. Previously, we have demonstrated that LTH leads to a 44% reduction in Ins(1,4,5)P3 release by NE-induced α1-AR activation [18]. In the present study, we show that LTH is also associated with an increased involvement of PKC in α1-AR induced contractility. Of note, LTH is known to suppress calcium-dependent mechanisms and increase reliance of calcium-sensitization mechanisms [24], [36].

We also have shown that ERK1/2 negatively regulate PKC-induced contractility and play a major role in calcium-sensitization pathway [23]. In the present study, we also demonstrate that PHE-induced contractility is increased significantly by inhibition of ERK phosphorylation (Figure 10). Inhibition of α1B-AR, did not produce an increase in PHE-induced contractility by ERK inhibitors. This finding suggests that PHE activates α1B-AR which (may be via PKC) activates ERK, which in-turn negatively regulates arterial contractility. Another important pathway, which can be activated by PKC is of Rho kinase [37]. Of note, inhibition of Rho kinase by Y-27632, completely inhibited PHE-induced contractile responses in both normoxic and LTH acclimatized sheep MCA. This is in agreement with a previous study demonstrating a significant inhibition of PHE-induced contractile response in rat-tail artery [38]. Thus, it appears that Rho kinase is critically involved in PHE-induced cerebral arterial contractility.

The role of specific α_1_-AR subtypes in the regulation of cerebrovascular tone and cerebral blood flow in response to LTH acclimatization is not well understood. A normal acclimatization response is associated with an increase in hematocrit, hemoglobin concentration, and capillary density along with normal cerebral blood flow. However, the regulatory pathways maintaing normal (as in sea-level control) cerebral blood flow despite reduced cardiac output are not completely understood. In recent years, studies have demonstrated that adrenergic system assumes an important role in the maintenance of the cerebral blood flow. Moreover, unlike other systemic circulatory beds, large arteries have been shown to play a crucial role in the regulation and maintenance of CBF [39]. During increased flow demand, there is a significant pressure gradient from carotid to cerebral arteries [40]. Additionally, other studies suggest that much of the change in systemic pressure results in dilation/contraction of the large arteries that supply the brain [41]. These studies underscore the importance of middle cerebral artery in the regulation of CBF, and suggest that failure of middle cerebral artery to effectively regulate the pressure of the blood reaching delicate intracerebral and plial arteries may lead to their rupture with hemorrhage. This is of vital importance, as the α_1_-AR subtypes play a role in regulation of large cerebral arteries. Quite obviously, we yet have much to learn about α_1_-AR subtypes and their changing role with LTH acclimatization. A caveat of these studies concerns the relative selectivity of the purported pharmacologic agonist/antagonists for the several α_1_-AR subtypes, as there may be some limited interaction with other adrenergic and non adrenergic receptors. Nonetheless, in previous studies, these agents have been demonstrated to be quite selective antagonists for α_1_-AR subtypes [12], [19], [31]–[33].

## Perspective and Significance

**Figure 11:**
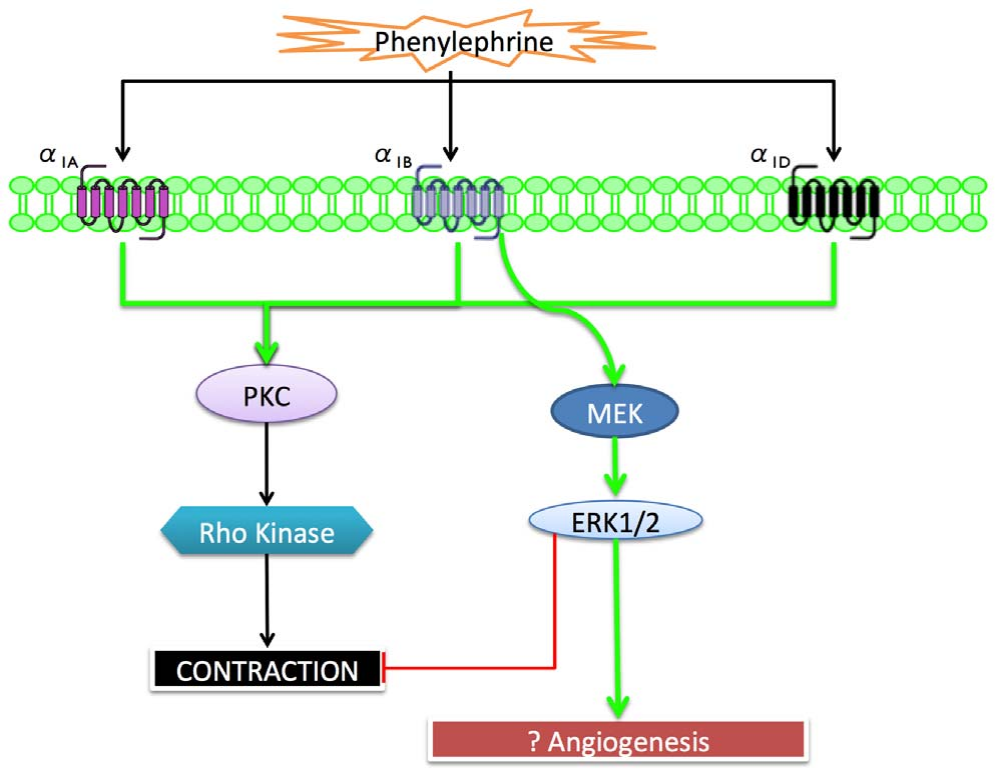
Proposed signal transduction pathways for specific α_1_-AR subtype-mediated MCA contraction in MCA.

The present study extends our understanding of the ovine adrenergic system in the contractility of MCA and ultimately regulation of the cerebral blood flow. It is a logical extension of our previous findings and illustrates a small facet of this complexity. The findings provide details regarding LTH-induced acclimatization in expression and functions of the α_1_-AR subtypes, as well as their coupling to downstream pathways. For a mechanistic overview, Figure 11 presents a schema based on our present understanding of the role of specific α_1_-AR subtype-mediated mechanisms in ovine MCA. An important take home message of the present study is LTH-induced increase in α1B-AR expression and their role in ERK activation. Nonetheless, this study makes apparent the complexity of intracellular signaling pathways and raises a number of important questions. For instance, by what mechanisms does LTH increase α1B-AR expression? What is the functional significance of increase in α_1B_- AR with LTH? To what extent is α1B-AR-mediated ERK activation responsible for vascular growth and/or other gene expression? What other signaling pathways are involved in α_1_-AR-mediated contractile responses in adult ovine MCA? Also of importance, how can we explain the paradox of decreased PHE-induced contractility despite an increase or no change in α1-AR subtype levels? This suggests acclimatization to LTH may be associated with changes in the plasma membrane receptor localization or internalization, or changes in the other signal transduction pathways. Insight into these pathways and physiologic mechanisms may prove to be of great clinical value in developing therapeutic interventions to prevent and/or ameliorate the sequelae of functional dysregulation of cerebral blood flow at high altitude and other conditions associated with LTH exposure.

## Supporting Information

Figure S1 **Relative mRNA levels (A) Glyceraldehyde 3- Phosphate Dehydrogenase and (B) Beta Actin in normoxic and LTH cerebral arteries by real-time PCR.** n = 5 sheep in each group. Values are means ± standard error of means. *Denotes P = <0.05. Fold change was relative to 18 s Ribosomal RNA. GAPDH - Glyceraldehyde 3- Phosphate Dehydrogenase; ACTB - Beta Actin.
(TIFF)

Figure S2 **Picture of RNA integrity gel demonstrating two distinct bands of 28** **s and 18** **s RNA bands.** Samples 1 to 5 represents RNA isolated from normoxic cerebral arteries, whereas samples 6 to 10 represents those from LTH acclimatized sheep.
(TIFF)
