# Correction: Cerebral Artery Alpha-1 AR Subtypes: High Altitude Long-Term Acclimatization Responses

**DOI:** 10.1371/journal.pone.0123491

**Published:** 2015-04-02

**Authors:** The PLOS ONE Staff

Fig. 4C incorrectly appears as a duplicate of Fig. 2C. The authors have provided a complete, correct version of Fig. 4 here.

**Fig 4:**
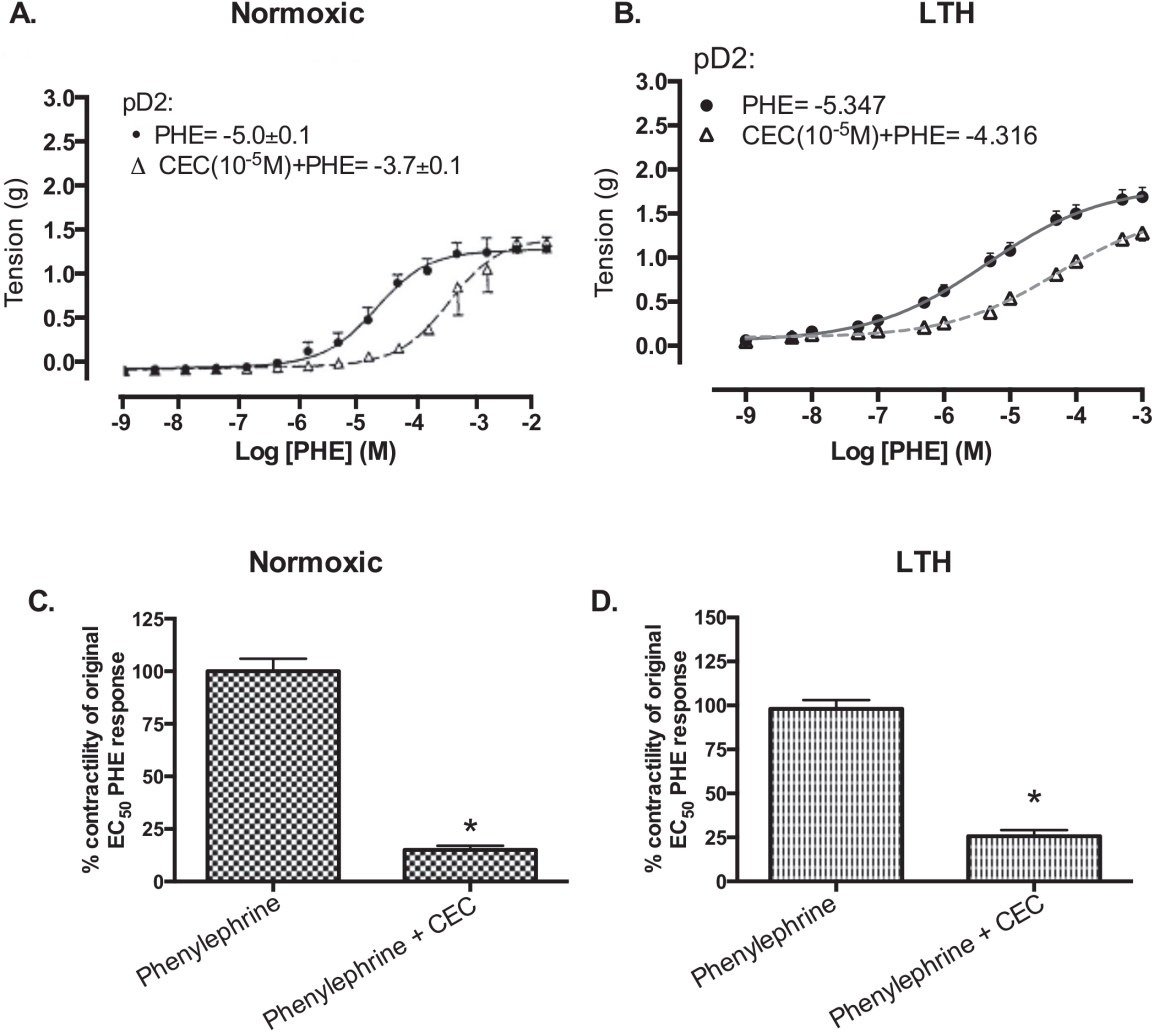
PHE responses in presence and absence of α1B-AR subtype antagonist (10^−5^ M CEC) in normoxic and LTH ovine MCA. (A) Dose-response curves in normoxic MCA. Vascular tensions (g) for MCA in response to PHE alone (•, solid line), and in the presence of 10^−5^ M CEC (Δ, dashed line). (B) Dose-response curves in LTH MCA. (C) Tension (g) at EC50 in normoxic sheep MCA. (D) Tension (g) at EC50 in LTH sheep MCA. n = 5 sheep in each group. Values are means ± standard error of means. *Denotes P = <0.05.
