# Supplementary figures and images for: Cerebral Artery Alpha-1 AR Subtypes: High Altitude Long-Term Acclimatization Responses
Source: PLoS One. 2014 Nov 13;9(11):e112784. doi: 10.1371/journal.pone.0112784 (PMC4231100; doi:10.1371/journal.pone.0112784)

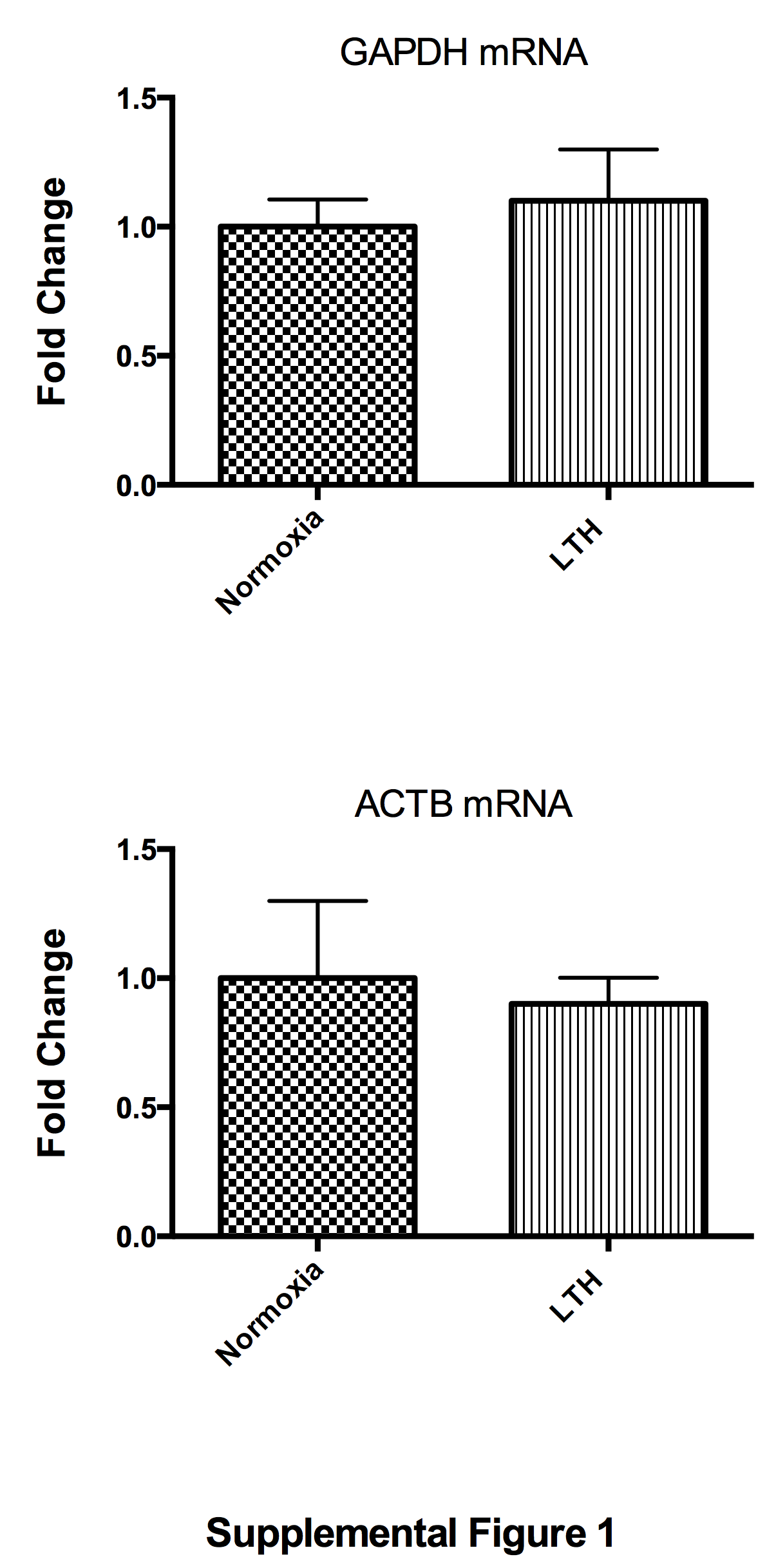

Supplement: Figure S1 — Relative mRNA levels (A) Glyceraldehyde 3- Phosphate Dehydrogenase and (B) Beta Actin in normoxic and LTH cerebral arteries by real-time PCR. n = 5 sheep in each group. Values are means ± standard error of means. *Denotes P = <0.05. Fold change was relative to 18 s Ribosomal RNA. GAPDH - Glyceraldehyde 3- Phosphate Dehydrogenase; ACTB - Beta Actin. (TIFF) [file pone.0112784.s001.tiff]

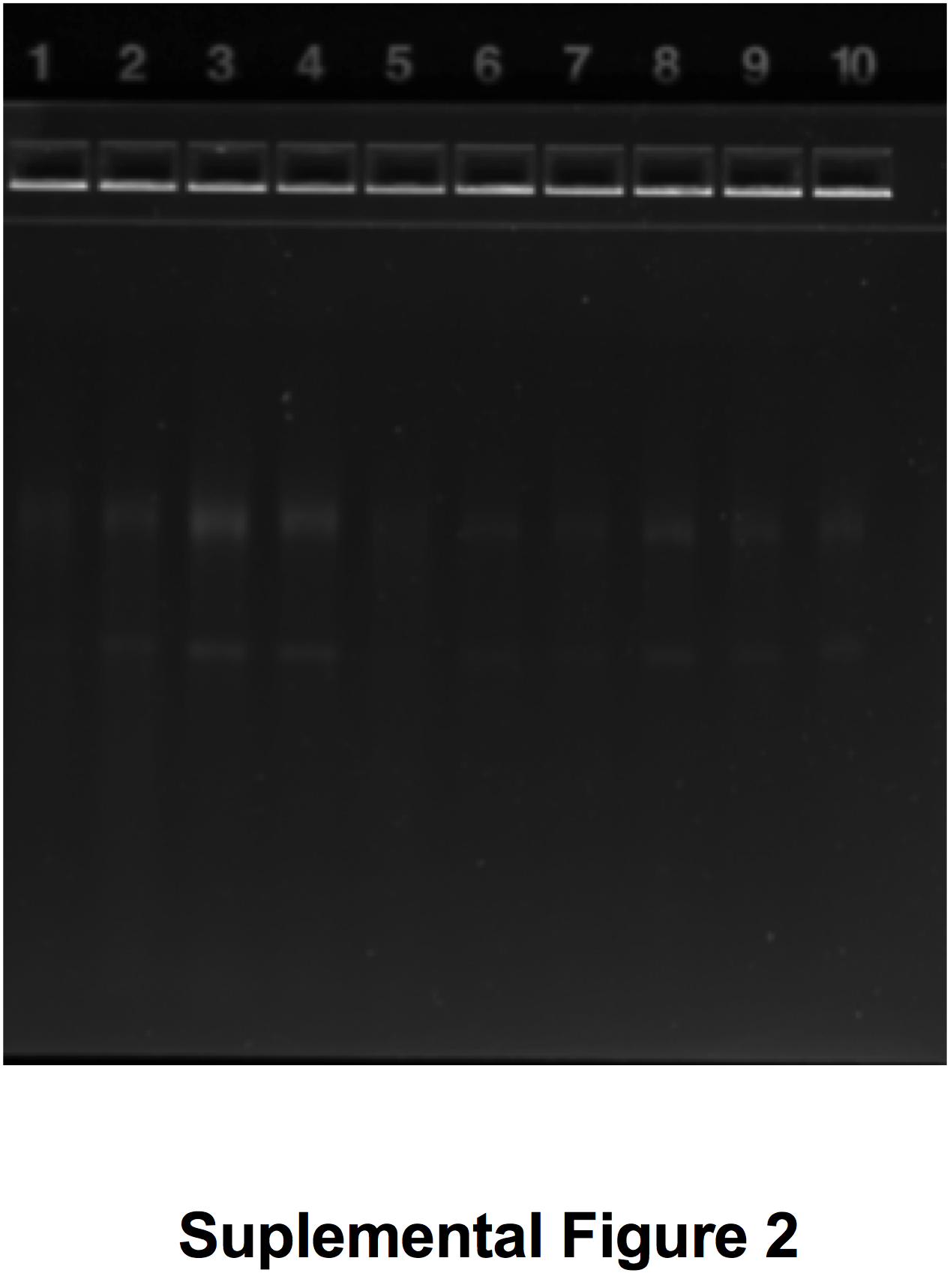

Supplement: Figure S2 — Picture of RNA integrity gel demonstrating two distinct bands of 28 s and 18 s RNA bands. Samples 1 to 5 represents RNA isolated from normoxic cerebral arteries, whereas samples 6 to 10 represents those from LTH acclimatized sheep. (TIFF) [file pone.0112784.s002.tiff]
